# Fatigue as a central bridge: temporal dynamics between problematic smartphone use and depressive symptoms in Chinese adolescents

**DOI:** 10.1186/s13034-025-00937-x

**Published:** 2025-07-05

**Authors:** An’an Hu, Yan Zhang, Jiaxing Sun, Xiubin Wang, Misaki N Natsuaki, Nengzhi Jiang

**Affiliations:** 1 School of Psychology, Shandong Second Medical University, 7166 Baotong West Street, Weifang, 261053 Shandong China; 2 Department of Psychology, University of California, 900 University Avenue, Riverside, Riverside, CA 92521 USA

**Keywords:** Problematic smartphone use, Depressive symptoms, Adolescents, Cross-lagged panel network analysis

## Abstract

**Background:**

The prevalence of problematic smartphone use (PSU) has been increasing among adolescents in recent years, often co-occurring with depressive symptoms, which poses additional challenges to adolescent mental health. Despite growing concern, the mechanisms underlying the co-occurrence of PSU and depression remain poorly understood. To address this gap, the current study employed cross-lagged panel network analysis to investigate the temporal relationships between specific symptoms of PSU and depressive symptoms over time.

**Methods:**

Data were collected at three time points (T1, T2, and T3), with six-month intervals between each wave. Participants self-reported their levels of depressive symptoms and PSU. A total of 558 participants (52.5% male; mean age at T1 = 13.83, SD = 0.78) were included in the final analysis. Two cross-lagged panel networks were constructed to examine the bidirectional relationships between depressive symptoms and PSU from T1 to T2 and from T2 to T3.

**Results:**

In the T1-T2 network, *Withdrawal* from PSU and *Fatigue* from depressive symptoms not only emerged as the most influential symptoms but also acted as bridge symptoms linking the co-occurrence of these two mental health issues. In the T2-T3 network, the structure of network became denser, with the most influential symptoms primarily stemming from depressive symptoms, such as *Sleep Disturbance* and *Feeling of Failure*. *Negative Life Consequences* from PSU and *Fatigue* from depressive symptoms served as key bridge symptoms.

**Conclusions:**

The findings provide valuable insights into the temporal dynamics underlying the co-occurrence of PSU and depressive symptoms during adolescence, with Fatigue appearing to play a significant role in linking these two mental health issues over time. Future studies should account for individual differences in how symptoms evolve over time and explore how these symptoms develop and persist at the individual level.

**Supplementary Information:**

The online version contains supplementary material available at 10.1186/s13034-025-00937-x.

## Background

With the continuous development of technology and digitalization, smartphones have become an indispensable part of daily life. Smartphones have revolutionized many aspects of adolescent life, including influencing their education (e.g., accessing information for schoolwork via search engines, participating in online learning, and completing assignments), social interactions (e.g., maintaining friendships and building connections through social media and messaging apps), and leisure activities (e.g., streaming videos and music, playing online and offline games) [1]. Changes in social environments and life patterns have made adolescents increasingly dependent on smartphones, raising significant concerns about problematic smartphone use (PSU).

PSU is widely recognized as an individual’s inability to regulate the usage of smartphones, resulting in negative consequences and functional impairments in daily life, such as decreased academic performance, impaired social relationships, reduced sleep quality, attention difficulties, and increased mental health issues like anxiety and depression [1–5]. It is often accompanied by symptoms such as craving, tolerance, withdrawal, and subjective loss of control [6, 7]. A meta-analysis concluded that the global prevalence of PSU among children and young people is as high as 23.3% [8] and 23.5-52.8% in China, specifically [9–12]. A large number of studies have consistently found that PSU is significantly associated with a range of mental health problems, including poor sleep quality [5], depression [1, 5, 13–15], anxiety [15, 16], and chronic stress [17, 18].

Adolescence is a dynamic phase of life characterized by rapid physiological development and gradual psychological maturity [19], which often give rise to notable emotional and behavioral challenges [20]. Research has shown that adolescents are not only susceptible to PSU due to their inadequate ability of self-control [21, 22] but are also at risk of experiencing the onset of depression [23], which increases the risk for depression in adulthood [24].

Depression, which is estimated to be as high as 24.3% among Chinese adolescents [25], is frequently observed co-occurring with PSU [26, 27]. For example, a meta-analysis indicated that PSU may serve as both an indicator of anxiety and depressive symptoms and a potential manifestation of these mental health issues in modern society [28]. Additionally, a previous longitudinal study found that depression was a salient predictor of PSU [29]. Moreover, other longitudinal studies have demonstrated that PSU and depressive symptoms are bidirectionally related [30, 31]. Therefore, it is necessary to explore the mechanisms behind the occurrence of these two conditions so that more effective interventions can be delivered.

Unfortunately, despite a large body of research demonstrating the co-occurrence of these two conditions, the mechanisms underlying it still remain disputed. Elhai and Dvorak proposed three mainstream explanations [17]. The first model suggests that mental health problems (such as depressive symptoms) can cause PSU. According to this model, individuals facing negative emotions in real life might seek to use their phones to escape real life problems [32, 33]. This hypothesis has received empirical support [34, 35], which aligns with the compensatory internet use theory [36]. The second model hypothesizes that indulging in smartphone use can cause depressive symptoms. This hypothesis, which is rooted in the social displacement hypothesis [33, 37], posits that excessive smartphone use reduces individuals’ engagement in offline activities, resulting in social isolation, which in turn can contribute to the development of depressive symptoms [38, 39]. The third hypothesis is the bidirectional reinforcement model, which suggests that there exists a bidirectional relationship between the two conditions, where PSU contributes to the development of depressive symptoms, and in turn, depressive symptoms further exacerbate problematic smartphone use [17, 39, 40]. For instance, individuals experiencing PSU may spend excessive time on their phones, resulting in reduced offline social interactions and disrupted sleep patterns. Over time, this may contribute to a reluctance to engage with others and the development of sleep-related issues, ultimately leading to the emergence of depressive symptoms. In turn, individuals may increasingly rely on phone usage as an escape from real-world pressures, further exacerbating PSU and giving rise to a vicious cycle intertwined with depressive symptoms [32]. This perspective effectively aligns with the network analysis theory, a novel approach to understanding psychopathology [41–43].

Network analysis has gained acceptance as a new approach to understanding psychopathology and has been applied in the study of various conditions, including depression [44–46], comorbidity of anxiety and depression [47, 48], PTSD [49, 50], and internet addiction [26, 33, 51]. A cross-sectional study on PSU and depression among Chinese university students found that *Negative Moods* and *Concentration Problems* served as bridge symptoms, linking both negative emotions and PSU [52]. Another cross-sectional study about internet addictions and depression among adolescents identified *Preoccupation with the Internet* and *Guilty* as core symptoms, with *Anticipation for Future Online Activities* as a bridge symptom [26]. However, a common limitation of these two studies is that they were both cross-sectional. This design allows for the identification of partial correlations between variables but does not provide insights into the directionality of relationships or the dynamic changes in symptoms over time.

Responding to the above issues, Rhemtulla and his colleagues introduced the cross-lagged panel network (CLPN), which combines the cross-lagged panel model with network analysis model [53]. In CLPN, the focus is on examining high out-degree central symptoms (predicting other symptoms) and high in-degree central symptoms (being predicted by other symptoms) [50]. The centrality measures used for this purpose are out-expected influence (Out-EI, the total values of outgoing edges connected to a symptom) and in-expected influence (In-EI, the total values of incoming edges connected to a symptom). Symptoms with high Out-EI drive the development of the disorder and thus represent potential targets for clinical intervention [54]. A longitudinal study on short video addiction and depressive symptoms among college students using CLPN suggested that a specific addiction symptom, *Tolerance*, and depressive symptom, *Anhedonia*, were found to be significant predictors of the subsequent development of symptoms for each mental health issue [55]. Moreover, the addiction-related issue of *Conflict* and emotional-related such as *Sad Mood* may function as bridge symptoms that connect the co-occurrence of these two mental health problems.

Although previous studies have provided in-depth insights into the relationship between PSU and depressive symptoms, there are several areas that still require further improvement and refinement. First, most research to date has focused on college student populations, leaving adolescence unexamined. Network construction is a dynamic process, and there is high heterogeneity in the core symptoms and key bridge symptoms displayed by different populations. In particular, adolescence, as a crucial period influencing individuals’ lives, is not only a key stage for physical and psychological development but also a peak period for the onset of mental conditions [20, 56]. Second, the majority of network analysis studies have been conducted using the cross-sectional framework. These networks lack directional edges, which limits the exploration of mutual predictive relationships between symptoms. Consequently, it is difficult to determine whether these central symptoms are more likely to trigger other symptoms or be triggered by them. To capture the evolving characteristics of symptoms at different stages and assess their predictive ability, it is necessary to employ CLPN and compare data collected at different time points. In order to fill these gaps in the literature, we collected three-wave longitudinal data in two secondary schools and applied CLPN to explore the association between PSU and depressive symptoms over time among adolescents. Therefore, the objectives of this study are to: (a) uncover the pathways between PSU and depressive symptoms and identify the edges with the strongest connections; (b) identify the Out-EI symptoms and In-EI symptoms in the T1-T2 and T2-T3 CLPN to assess their predictive power; and (c) identify the BEI symptoms that connect these two mental health issues.

## Method

### Participants and procedure

The current study was conducted as a longitudinal study using cluster sampling in two secondary schools in Shandong, China, targeting first- and second-year students as the survey participants. We acquired permission from the schools’ headmasters and informed consent from participants and their parents before the survey was conducted.

We collected three waves of data, each with an interval of six months between data collection. Researchers provided instructions and administered the survey, and the students completed questionnaires in the classroom. All students were informed that they could withdraw from the survey at any time. The questionnaires were collected by the researchers on-site after all participants had completed them. The administration procedures were identical across the three time points. This study received approval from the Research Ethics Board of the university affiliated with the corresponding author (2021YX027).

We retained data for students who participated in all three survey waves by matching names across the datasets. In this dataset, we excluded samples with more than 50% missing data and applied multiple imputation to handle missing values in the remaining samples. Eventually, a total of 558 participants (52.5% male; mean age at T1 = 13.83 years, SD = 0.78, age range = 12 to 15 years) were included in the final analysis. Information about the demographic characteristics of the final sample at T1 is presented in Table 1.

**Table 1:** Description of demographic characteristics (numbers and percentages) at T1

Variables	*N (%)*
Grades	
7th Grade	305 (54.7%)
8th Grade	253 (45.3%)
Gender	
Male	293
Female	265
Educational level (Father)	
Primary School or Below	27
Junior High School	344
Senior High School or Vocational School	142
Associate Degree	31
Undergraduate and above	14
Educational level (Mother)	
Primary School or Below	37
Junior High School	356
Senior High School or Vocational School	129
Associate Degree	20
Undergraduate and above	16

### Measures

*Depressive symptoms* were assessed using the Chinese version of the nine-item Patient Health Questionnaire (PHQ-9) [57, 58]. The scale ranged from 0 (“not at all”) to 3 (“nearly every day”) to assess the participants’ condition of depression over the past two weeks. Higher scores reflect a higher level of severity in depression. This study showed good reliability for the PHQ-9, with Cronbach’s α values of 0.899, 0.889, and 0.941 at T1, T2, and T3, respectively.

*The problematic smartphone use* was assessed using the Chinese version of 10-item Mobile Phone Problem Use Scale (MPPUS-10) [59]. The scale includes five dimensions: Negative Life Consequences, peer acceptance, craving, withdrawal, and loss of control [14, 60]. In the current study, the dimensional subscales of the MPPUS-10 were averaged to consolidate the scale into five dimensions, each represented by an average score. The original MPPUS-10 employs a Likert scale ranging from 1 (“not true at all”) to 5 (“extremely true”). After the scale was condensed, the total score ranged from 0 to 25, with higher scores indicating greater severity of PSU. In this study, the Cronbach’s α values for the MPPUS-10 were 0.901, 0.897, and 0.929 at T1, T2, and T3, respectively.

### Statistical analysis

Descriptive statistics of demographic information, and mean (M), standard deviation (SD) of all the items in the PHQ-9 and MPPUS-10 were calculated by SPSS (Version 26.0). The R (Version 4.3.3) was applied to perform CLPNs [53].

In network analysis, the term “community” commonly refers to a group of psychological variables [27, 55, 61]. Within this framework, symptoms are defined as nodes, and the associations between symptoms are defined as edges [41]. Central symptoms, which usually have the highest centrality, are considered to have the most important impact on the entire symptom network [42, 53, 62]. The expected influence (EI) is deemed to be a solid centrality index, for it preserves the positive and negative signs of the edge weights before summing them [43, 62]. Bridge symptoms are nodes with the highest bridge centrality and serve as connectors between two conditions [61, 63].

*CLPNs estimation.* We estimated two CLPNs from T1 to T2, and T2 to T3. To estimate CLPNs, both autoregressive effects (a symptom at T1 predicts itself at T2) and cross-lagged effects (a symptom at T1 predicts other symptoms at T2) were calculated using a series of logistic regressions. The students’ age, gender, and parental educational level (both father and mother) were included in the analysis as covariates. A previous study suggested the cross-validation is preferable for selecting network model [64], for which it had the lowest specificity (also called as True Negative Rate, TNR) and the highest sensitivity (True Positive Rate, TPR) [53]. The R package “glmnet” [65] was applied to regularize regressions using LASSO (Least Absolute Shrinkage and Selection Operator) [66] with 10-fold cross-validation to shrink the small value edges to zero, so that a more sparse and accurate network structure could be presented. In order to achieve better interpretability, we transformed the regression coefficients (i.e., edge weights) into odds ratios (ORs) by exponentiation. The value of edge weights equal to 1 indicates there is no correlation between two nodes. The value higher than 1 indicates a positive connection, and the value lower than 1 shows a negative connection [64]. The plots of CLPNs were using the R package “qgraph” [66] to present.

*Centrality calculation*. Considering CLPN is directed, therefore, the In-EI and Out-EI were calculated to identify the directional predictability of nodes. The BEI was also calculated by the R package “networktools” to find out which nodes are bridge symptoms that play bridge roles between two communities. Higher values of BEI indicate an increased likelihood of contagion spreading to other communities, thus amplifying the potential risk.

*Network accuracy and stability.* To assess the accuracy and stability of the CLPNS, we used the R package “bootnet” [67]. First, we calculated the 95% confidence intervals (CIs) of all edges by using the non-parametric bootstrap method (with 1,000 permutations) to evaluate the accuracy of edge weights. Narrower CIs indicate a more accurate CLPNs’ structure. Second, the stability of correlations was assessed by using the case-dropping bootstrap method (with 1,000 permutations). The values of the method (correlation stability coefficient, CS-coefficient) represent the maximum proportion of the sample that can be removed without causing a significant change to the original data. We also tested the edge weights and node centralities to identify whether there were statistical significances. The CS - coefficient above 0.5 is considered to have an ideal interpretability and stability, and under 0.25 is considered unacceptable [67]. Furthermore, the Jaccard Index was calculated to show the consistency of the two CLPNs, and higher value indicated a more consistent structure [68].

## Results

### Descriptive analysis

The mean total scores of PHQ-9 were 4.28 (SD = 4.93) at T1, 2.42 (SD = 3.80) at T2, and 3.50 (SD = 4.71) at T3. The mean total scores of the MPPUS-10 were 11.23 (SD = 3.92), 11.74 (SD = 3.82), and 11.61 (SD = 4.18) across the three time points. Table 2 presents all symptoms along with their corresponding labels, the community to which each symptom belongs, and their means and standard deviations.

**Table 2:** Symptom labels, communities and descriptive statistics (Mean and Standard deviation) at three time points (N = 558)

Symptom	Label	Community	T1		T2		T3	
			M	SD	M	SD	M	SD
Anhedoina	P1	Depressive Symptoms	1.46	0.73	1.30	0.58	1.43	0.65
Depressed Mood	P2	Depressive Symptoms	1.41	0.69	1.23	0.53	1.39	0.65
Sleep Disturbance	P3	Depressive Symptoms	1.59	0.86	1.40	0.71	1.46	0.71
Fatigue	P4	Depressive Symptoms	1.62	0.79	1.34	0.64	1.48	0.66
Appetitive Disturbance	P5	Depressive Symptoms	1.45	0.69	1.28	0.61	1.40	0.65
Feeling of Failure	P6	Depressive Symptoms	1.58	0.81	1.25	0.57	1.38	0.64
Concentration	P7	Depressive Symptoms	1.66	0.82	1.38	0.69	1.46	0.68
Motor	P8	Depressive Symptoms	1.27	0.59	1.13	0.39	1.29	0.53
Suicidal Ideation	P9	Depressive Symptoms	1.25	0.59	1.12	0.39	1.21	0.49
Craving	M1	Problematic Smartphone Use	2.98	1.11	3.08	1.10	2.99	1.12
Withdrawal	M2	Problematic Smartphone Use	1.83	0.87	1.91	0.86	1.97	0.92
Negative Life Consequences	M3	Problematic Smartphone Use	1.63	0.74	1.73	0.79	1.76	0.80
Loss of Control	M4	Problematic Smartphone Use	2.36	1.05	2.46	1.02	2.37	1.10
Peer Dependence	M5	Problematic Smartphone Use	2.43	1.19	2.57	1.17	2.53	1.16

### Network accuracy and stability

**Fig. 1:**
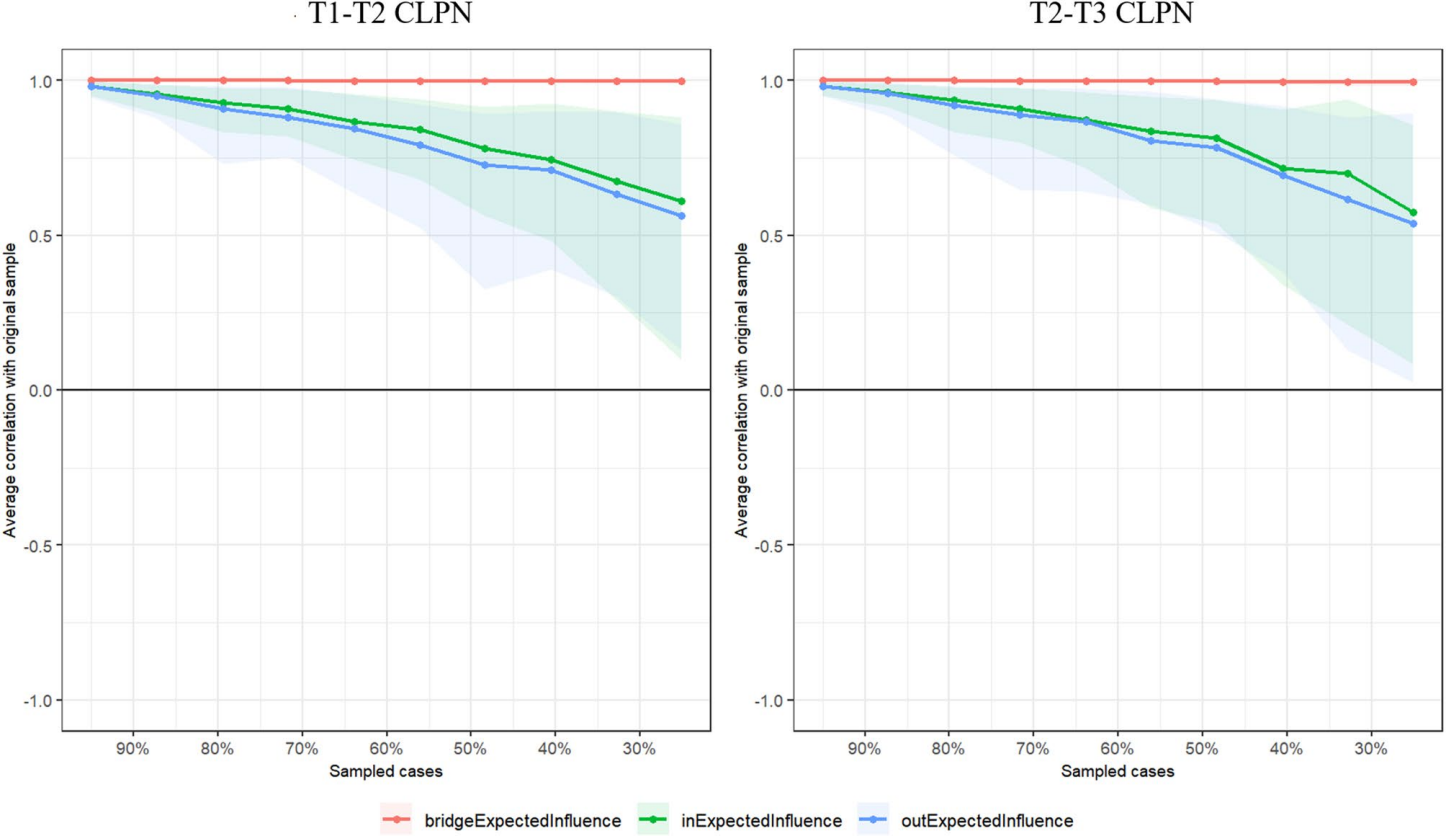
Stability of centrality indices by case dropping subset bootstrap. The x-axis shows the percentage of cases from the original sample used at each step, while the y-axis displays the average correlations between centrality indices from the original network and those from networks re-estimated after progressively dropping cases. Each line represents correlations for BEI, In-EI and Out-EI, with shaded areas indicating the 95% confidence intervals

As in Fig. S1, the 95% CIs of all edge weights using the bootstrap method were presented, and the CIs of edge weights were small to moderate. We calculated centrality indices using the case-dropping bootstrap method and found out that the BEI had greatest stability, Out-EI and In-EI had moderate stability (BEI’s CS-coefficient = 0.749, Out-EI’s CS-coefficient = 0.283, and In-EI’s CS-coefficient = 0.439 in the T1-T2 CLPN structure; in the T2-T3 CLPN structure, BEI’s CS-coefficient = 0.749, Out-EI’s CS-coefficient = 0.362, and In-EI’s CS-coefficient = 0.439). Since all CS-coefficients exceeded the minimum threshold of 0.25 for acceptable stability [67], we included all three indices in the analysis (Fig. 1). We also performed the edge weights difference test and centrality difference test, see Fig. S2 and Fig. S3.

### CLPNs’ structure

**Fig. 2:**
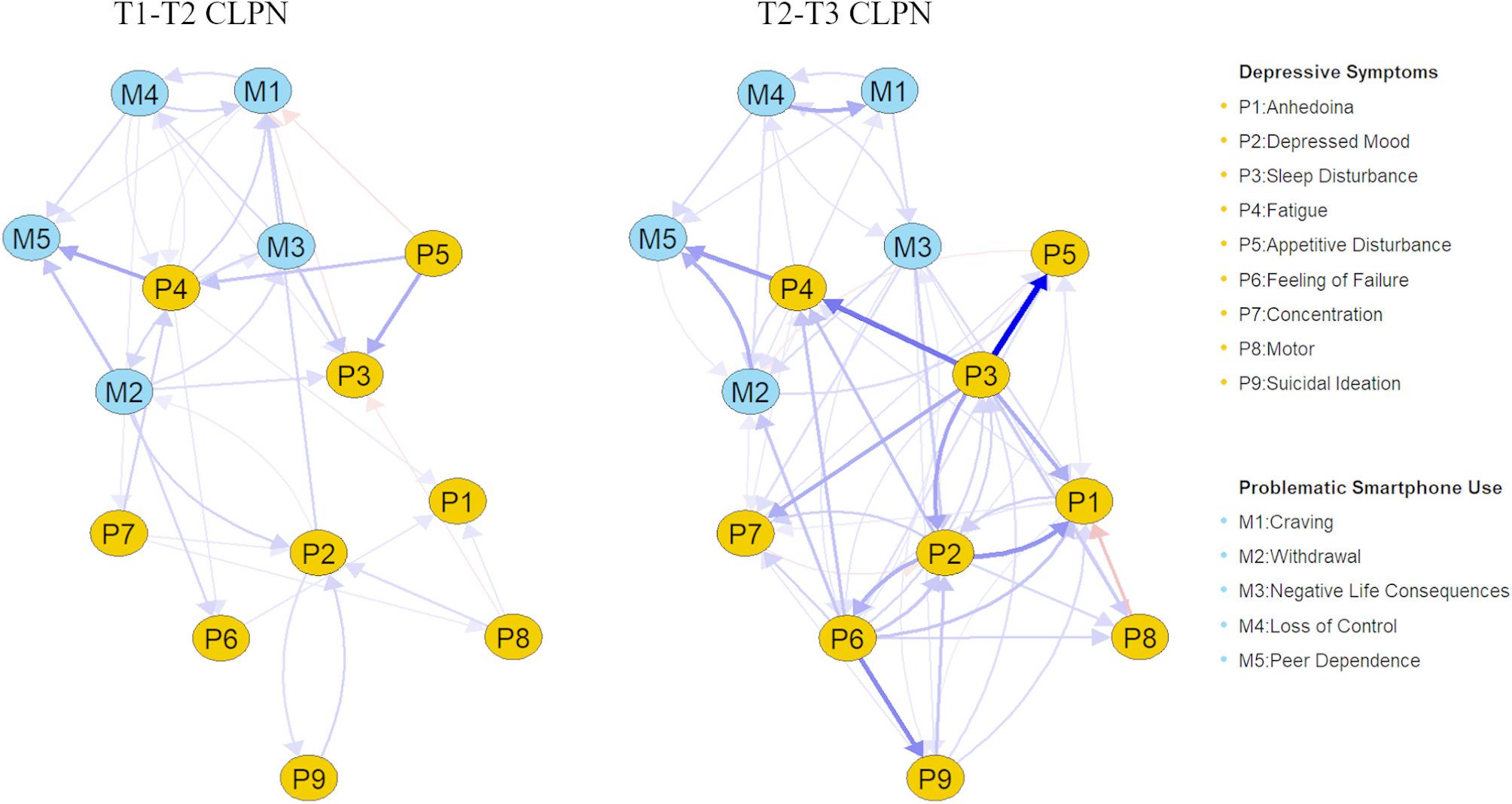
The cross-lagged panel networks for T1-T2 and T2-T3 (Thresholds = 0.05). Arrows represent the directional relationships. Blue edges indicate positive relationships (i.e., odds ratios greater than 1), and red edges indicate negative relationships (i.e., odds ratios less than 1). Edge thickness represents the weight of the odds ratios such that thicker edges represent stronger relations. To simplify visual interpretation, autoregressive edges, weaker edges (i.e., odds ratios within 1 ± 0.05), and covariates were excluded from the plot

The values of edge weights in ORs are presented in Tables S1 and S2. The mean ORs of autoregressive edges were 1.22 in the T1-T2 CLPN, 1.25 in the T2 -T3 CLPN. Regarding the cross-lagged edges, the mean ORs were 1.02 for T1-T2 CLPN and 1.04 for T2 -T3 CLPN. The purpose of presenting the structure of the CLPNs is to highlight the strongest paths, and thus, we excluded autoregressive edges to improve the visual interpretability of the cross-lagged edges in Fig. 2. The none zero edges in the T1-T2 CLPN were 89, 115 in the T2-T3 CLPN. In order to create a sparse structure, the edges that with ORs within 1 ± 0.05 were also removed from Fig. 2 [55]. The plots that contain all the edges and autoregressive edges are presented in Fig. S4. Following the recommendations of Jones and prior network analysis studies, we adopted a blind 80th percentile threshold (i.e., top 20%) to identify the most influential nodes and strongest edges [55, 61, 69]. Based on this criterion, in the T1-T2 CLPN, the three strongest cross-lagged edges were *Fatigue* (P4) → *Peer Dependence* (M5; OR = 1.20), *Appetitive Disturbance* (P5) → *Sleep Disturbance* (P3; OR = 1.19), and *Appetitive Disturbance* (P5) → *Fatigue* (P4; OR = 1.15), which were all positive connections. The edge *Fatigue* (P4) → *Peer Dependence* (M5) also identified as the strongest connecting bridge edge. Additionally, another relatively strong bridge edge was observed: *Negative Life Consequences* (M3) → *Sleep Disturbance* (P3; OR = 1.14).

In the T2-T3 CLPN, the three strongest cross-lagged edges were all positive: *Sleep Disturbance* (P3) → *Appetitive Disturbance* (P5; OR = 1.33), *Sleep Disturbance* (P3) → *Fatigue* (P4; OR = 1.27), and *Feeling of Failure* (P6) → *Suicidal Ideation* (P9; OR = 1.25). The only bridge edge that had a strong connection was *Fatigue* (P4) → *Peer Dependence* (M5; OR = 1.22).

**Fig. 3:**
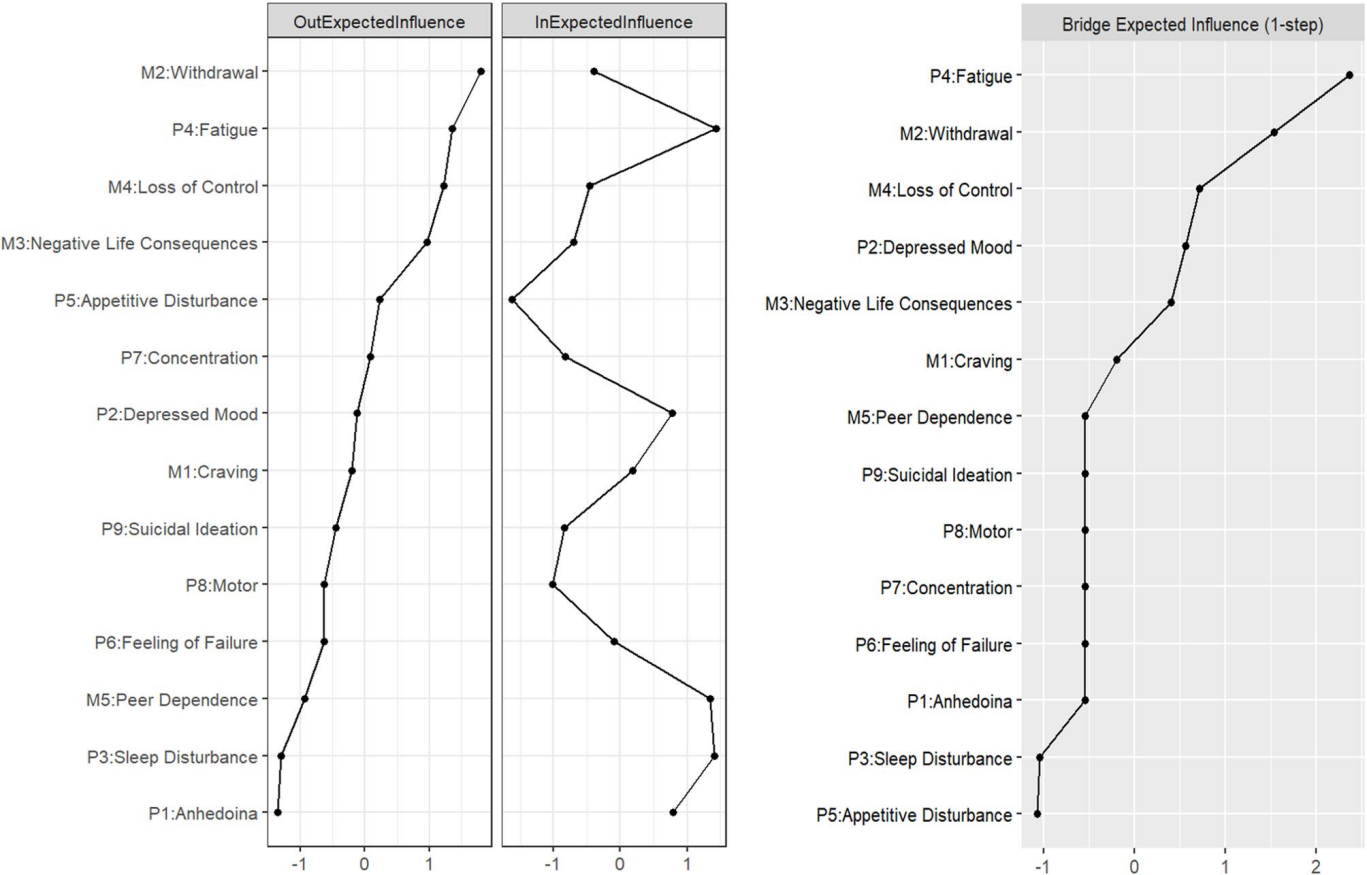
Centrality estimates and BEI in the T1-T2 CLPN. Larger values reflect greater centrality

Figure 3 presented the centrality estimates and BEI in the T1-T2 CLPN. The nodes that had the highest Out-EI were *Withdrawal* (M2), *Fatigue* (P4) and *Loss of Control* (M4), suggesting that these were the most predictive symptoms of other symptoms at T2. *Fatigue* (P4), *Sleep Disturbance* (P3) and *Peer Dependence* (M5) were the nodes with the highest In-EI, which indicates these symptoms were the most likely to be predicted by other symptoms. The highest BEI nodes were *Fatigue* (P4) and *Withdrawal* (M2).

**Fig. 4:**
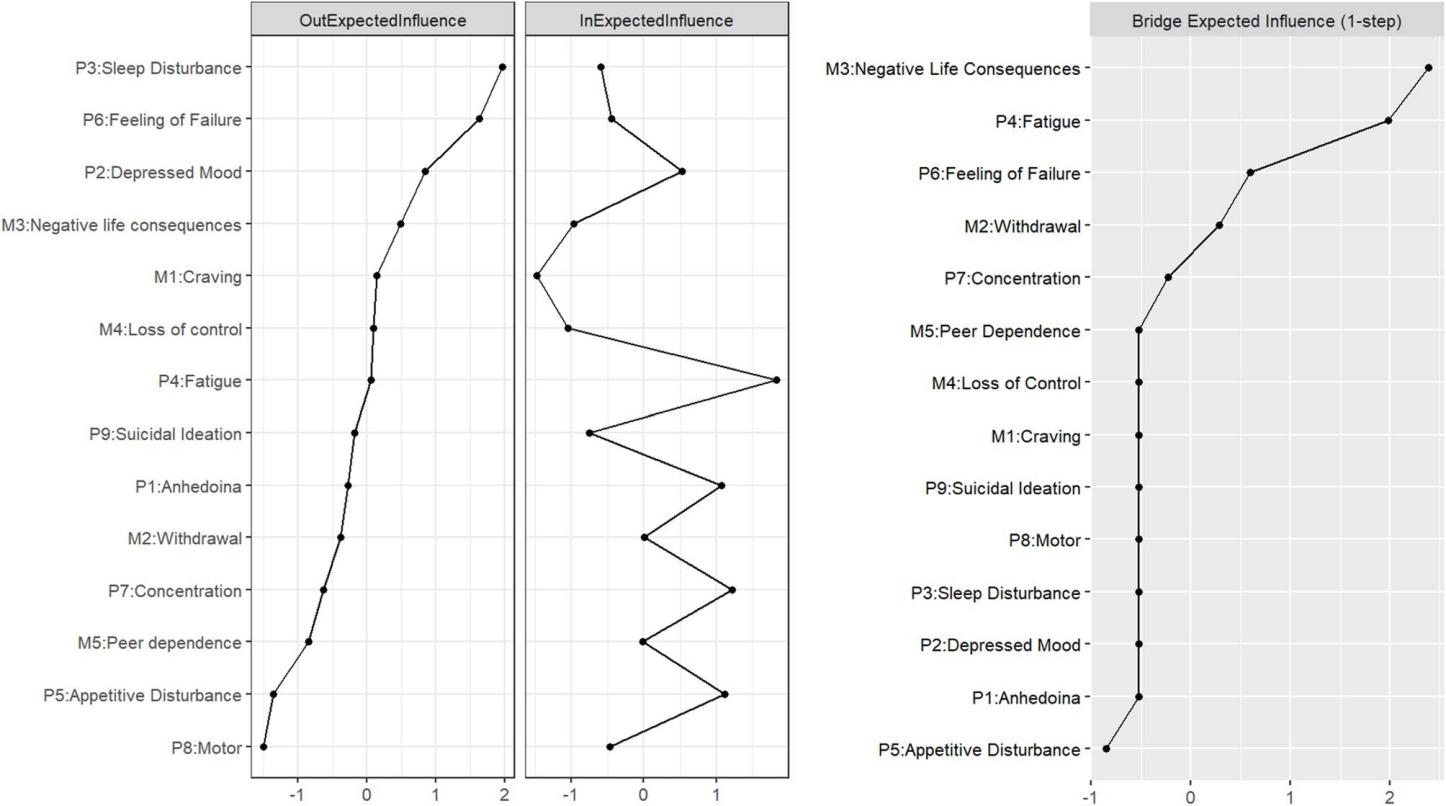
Centrality estimates and BEI in the T2-T3 CLPN. Larger values reflect greater centrality

Figure 4 displayed the centrality estimates and BEI in the T2-T3 CLPN. *Sleep Disturbance* (P3) and *Feeling of Failure* (P6) emerged as the nodes with the highest Out-EI, indicating that these symptoms were the most predictive of other symptoms at T3. Conversely, *Fatigue* (P4), *Concentration* (P7), and *Appetitive Disturbance* (P5) exhibited the highest In-EI, suggesting that these symptoms were the most influenced by others. Notably, the nodes with the highest BEI were *Negative Life Consequences* (M3) and *Fatigue* (P4).

## Discussion

The present study employed the CLPN approach on longitudinal data spanning three time points to examine the symptom-level dynamics, directional relationships, and bridge pathways between PSU and depressive symptoms among Chinese adolescents. Unlike prior cross-sectional networks analyses and cross-lagged panel models (CLPMs), CLPN enables a more detailed investigation into how specific symptoms influence one another over time. This approach allowed us to identify key symptoms and their evolving connections, offering deeper insight into the mechanisms underpinning the co-occurrence of PSU and depressive symptoms. To the best of our knowledge, this is the first study to apply temporal network analysis to this comorbidity in a Chinese adolescent sample, thereby advancing both methodological and contextual understanding in the field.

Specifically, in the T1-T2 CLPN, we found that *Withdrawal* (M2) from the PSU community and *Fatigue* (P4) from the depressive symptoms community not only had the highest Out-EI but also served as the strongest bridge symptoms. These findings stressed how important these two symptoms are in predicting the appearance of other symptoms and the connection between the two communities. Specifically, within this timeline of the PSU network framework, *Withdrawal* (M2) played a leading role and had a strong connection with *Peer Dependence* (M5). In the context of PSU, withdrawal typically refers to the impatience, anxiety, or irritability that individuals experience when their smartphone is inaccessible [70, 71]. The social aspect is one of the major reasons why adolescents use smartphones, and smartphones not only significantly altered their way of interacting with their peers but also their social relationships [72]. When adolescents are prohibited from using their smartphones, their ability to communicate is restricted, which may, in turn, increase their dependence on peers. As one of the bridge symptoms, *Withdrawal* (M2) had strong connections with *Depressed Mood* (P2), *Feelings of Failure* (P6), and *Sleep Disturbance* (P3), indicating that it can act as a trigger contributing to the co-occurrence of PSU and depressive symptoms. As a previous study demonstrated, *Depressed Mood* (P2), *Feeling of Failure* (P6) and *Fatigue* (P4) are the core symptoms of adolescent depression [73]. In line with the social displacement hypothesis, this may be due to students excessively engaging in smartphone use, which leads to sacrificing time for daily activities, potentially causing sleep disruptions, increasing negative feelings, and making them more vulnerable to depression [48]. The emergence of these core symptoms promotes and sustains the network of depressive symptoms. Additionally, the link between *Negative Life Consequences* (M3) and *Sleep Disturbance* (P3) also showed a strong connection, indicating that *Negative Life Consequences* from the PSU community, such as academic or social difficulties, contribute to poor sleep quality [5, 74]. Prior research has consistently shown that impaired sleep is both a consequence and a predictor of mental health problems in adolescents, particularly in the context of technology use [75–77]. Our findings further confirmed the predictive role of negative life consequences on sleep disturbances in adolescents, suggesting that life stressors in this group may exacerbate sleep problems.

Another key symptom and bridge symptom in the T1-T2 CLPN was *Fatigue* (P4). Similar to the previous study that focused on depressive symptoms among Chinese college students [68], we also found in our adolescent sample that *Fatigue* (P4) was the most influential symptom in triggering the activation of other depressive symptoms. Furthermore, *Fatigue* (P4), characterized by feelings of tiredness or low energy, is a common physical symptom of depressive disorder and is often considered one of the core symptoms in patients with clinical depression [78]. In the current study, *Fatigue* (P4) had strong connections with *Anhedonia* (P1). This finding is consistent with evidence from neuroscience suggesting that energy depletion (as seen in fatigue) may inhibit the nervous system’s ability to enhance the reception of rewards from positive stimuli, resulting in anhedonia [79, 80]. To link with the PSU network framework, *Fatigue* (P4) had an exceptionally strong connection to *Peer Dependence* (M5). One possible explanation is that when adolescents experience tiredness or low energy, they tend to seek companionship from their peers [81, 82].

Results from the T2-T3 CLPN indicated that *Sleep Disturbance* (P3) and *Feeling of Failure* (P6) had the highest Out-EI while *Negative Life Consequences* (M3) and *Fatigue* (P4) served as the highest bridge symptoms. As presented in Fig. 2, the T2-T3 CLPN was denser than the T1-T2 CLPN, especially within the depressive symptoms community.

In the T2-T3 CLPN, *Sleep Disturbance* (P3) was the most central symptom, and it had prominent connections with *Appetitive Disturbance* (P5) and *Fatigue* (P4) and strong connections with *Anhedonia* (P1), *Depressed Mood* (P2) and *Concentration* (P7). The onset of *Sleep Disturbance* (P3) can serve as a catalyst, initiating the development of related symptoms such as *Appetitive Disturbance* (P5) and *Fatigue* (P4). These subsequent symptoms contribute to the maintenance and exacerbation of depressive symptoms over time. Sleep-related problems are widespread among the adolescent population and have been identified as central factors in the development and maintenance of depression [83, 84]. Nowadays in China, students, in secondary school, are required to arrive at school early in the morning, which can lead to sleep deprivation. A previous study showed that just a modest (like 25 min) delay in school start time led to notable improvements in sleep duration, daytime sleepiness, and mood [85]. Due to their time being consumed by homework and parental control over phone usage, they are more likely to engage in retaliatory phone use during times when their parents are not watching (usually bedtime), thereby reducing their sleep time [30, 86]. Based on the issue, one possible solution is for parents to limit the amount of time adolescents spend on their phones and to take away their devices before bedtime.

*Feeling of Failure* (P6) was another central symptom in the T2-T3 CLPN and had an outstanding connection with *Suicidal ideation* (P9). *Feeling of Failure* (P6) is a culturally distinctive core symptom of depression among Chinese adolescents. Deeply influenced by Confucianism, Chinese parents’ well-documented enthusiasm for their children’s education is reflected in their exceptionally high expectations for academic performance, often regarding good grades as a critical pathway to a bright future and an essential indicator of future opportunities [73, 87]. However, high-achieving students are, after all, the minority. Academic stress is a risk factor for mental health, higher level of academic stress easily leads to a higher level of depression [88, 89].

*Negative Life Consequences* (M3) and *Fatigue* (P4) served as bridge symptoms that connected these two communities in the T2-T3 CLPN. *Negative Life Consequences* (M3), such as being late for school or experiencing poor academic performance due to excessive smartphone use, had striking links to *Depressed Mood* (P2) and *Anhedonia* (P1). A possible explanation is that some negative consequences resulting from smartphone use may create a sense of frustration in individuals, which in turn leads to negative emotions. However, the connection between *Negative Life Consequences* (M3) caused by PSU and depressive symptoms still remains unclear, future research should consider finding the mechanisms behind this connection.

Notably, although the central symptoms and bridge symptoms changed through time, the *Fatigue* (P4) as the bridge symptom remained same, and the edge that *Fatigue* (P4) pointed to *Peer Dependence* (M5) also had the highest bridge edge weights in both time stages. It suggests that depressive symptoms community mainly affect PSU through *Fatigue* (P4). The consistent bridging role of *Fatigue* may be due to its position at the intersection of behavioral and emotional dysregulation. Excessive smartphone use, particularly during nighttime, can disrupt sleep patterns and delay circadian rhythms, which in turn contribute to increased fatigue [90, 91]. Psychologically, fatigue may signal a depletion of emotional and cognitive resources, impairing motivation and concentration-key features of depressive symptoms [79, 92, 93]. In addition, psychological stress, such as academic or social pressures, may act as an underlying factor that exacerbates fatigue and reinforces its role as a bridge symptom [88, 89, 94, 95]. These findings suggest that targeting fatigue in interventions may be effective in mitigating the co-occurrence of PSU and depressive symptoms.

Overall, our findings supported and expanded the bidirectional reinforcement model, which posits that PSU and depressive symptoms co-develop through dynamic, mutually reinforcing feedback loops. This extension is consistent with patterns observed in other forms of problematic digital behavior and mental health [37, 55, 96]. Our study adds to this model by identifying a phase-specific directional pattern among Chinese adolescents: in the early stages, behavioral symptoms (e.g., PSU) primarily predicted emotional symptoms, whereas in later stages, emotional symptoms (e.g., depressive states) increasingly drive changes in behavior. This developmental trajectory offers new insights into how emotional and behavioral symptoms interact and mutually reinforce each other over time. While further research is needed to validate *Fatigue* as a primary intervention target, existing evidence-based interventions, such as mindfulness-based stress reduction (MBSR), have shown promise in reducing fatigue and emotional distress among adolescents [97, 98]. For instance, structured group activities such as mindfulness exercises and guided relaxation techniques can be integrated into class breaks or after-school programs. These approaches provide preliminary support for including fatigue-focused strategies in future intervention designs.

Several limitations should be considered when interpreting the findings of this study. First, the relatively short follow-up period of this study may not fully capture the long-term dynamics of depressive symptoms and PSU symptoms. These symptoms can fluctuate significantly over both short (e.g., hours or days) and long timeframes. Future studies should consider conducting longer-term follow-up with closer intervals and more advanced methods such as time-series network analysis to help uncover the interactions between these two conditions. Second, the current study used self-reporting method. Although self-reporting is a convenient method for assessing individual feelings and experiences, it is inherently subject to bias. Future studies should consider employing more reliable methods, such as multi-informant assessments, to strengthen the validity of the findings. Third, the participants in this study were drawn from two secondary schools in Shandong, China, which may limit the generalizability of our findings. Prior research suggests that both the manifestation of depressive symptom expression and patterns of smartphone use behaviors can vary across cultural and educational contexts [17, 99–101]. Therefore, future studies should include more demographically and culturally diverse samples to examine whether the co-occurrence patterns observed in this study are consistent across different populations. Fourth, the CLPN analysis identifies patterns at the group level, which may not reflect individual-level dynamics, underscoring the problem of ergodicity and individual heterogeneity [102–104]. Although this study reveals key group-level dynamics between problematic smartphone use and depressive symptoms among Chinese individuals, it remains unclear whether interventions targeting these nodes are clinically more effective. To address this, future research should collect denser longitudinal data and apply person-specific analytic approaches (e.g., Group Iterative Multiple Model Estimation) to evaluate the clinical utility of targeting these central variables [105]. Fifth, this study did not examine potential mediating or moderating variables, which may influence the co-occurrence between PSU and depressive symptoms. Prior research suggests that factors like parent–child relationship quality, physical activity, peer support, and emotion regulation can alter the strength and direction of this relationship [106–108]. Future studies should explore the role of these and other contextual variables to better elucidate the mechanisms underlying this co-occurrence.

## Conclusions

This is the first study that employed CLPN to investigate the longitudinal associations between PSU and depressive symptoms in Chinese adolescents. The findings strongly support the bidirectional model of these two mental health issues [32], with PSU playing a central role initially and influencing the onset of several depressive symptoms. In turn, these depressive symptoms further exacerbate PSU. The findings showed that during the T1-T2 time period, *Withdrawal* (M2) and *Fatigue* (P4) not only emerged as the most influential symptoms but also served as bridge symptoms that connect the PSU and depressive symptoms. During the T2-T3 time period, the structure of network became denser, particularly within the depressive symptoms community. In this phase, the most central nodes were *Sleep Disturbance* (P3) and *Feeling of Failure* (P6), while *Negative Life Consequences* (M3) and *Fatigue* (P4) served as bridge symptoms. Notably, *Fatigue* (P4) consistently served as a bridge symptom across both CLPNs, highlighting its potential as a key target for interventions aimed at preventing depressive symptoms and reducing the link between PSU and depressive symptoms in adolescents. Future studies should account for individual differences in how symptoms evolve over time and explore how these symptoms develop and persist at the individual level.

## Electronic supplementary material

Below is the link to the electronic supplementary material.

Additional file 1: Table S1. Adjacency matrix of the T1-T2 CLPN. Table S2. Adjacency matrix of the T2-T3 CLPN. Figure S1. Confidence intervals around edge weights for T1-T2 CLPN (left) and T2-T3 CLPN (right). Figure S2. Edge weight difference tests for T1-T2 CLPN and T2-T3 CLPN. Figure S3. Centrality difference tests for T1-T2 CLPN (a for Out Expected Influence, b for In Expected Influence) and T2-T3 CLPN (c for Out Expected Influence, d for In Expected Influence). Figure S4. The cross-lagged panel networks for T1-T2 (left) and T2-T3 (right) (including all autoregressive and cross-lagged edges).

## Data Availability

The datasets used and/or analyzed during the current study are available from the corresponding author on reasonable request.

## Abbreviations

PSU: Problematic smartphone use
EI: Expected influence
Out-EI: Out-expected influence
In-EI: In-expected influence
BEI: Bridge expected influence
CLPN: Cross-lagged panel network
SD: Standard deviation
M: Mean
PHQ-9: Patient Health Questionnaire-9
MPPUS-10: Mobile Phone Problem Use Scale-10
TNR: True negative rate
TPR: True positive rate
LASSO: Least absolute shrinkage and selection operator
OR(s): Odds ratio(s)
CIs: Confidence intervals
CS: Correlation stability
