# Supplementary material for: Fatigue as a central bridge: temporal dynamics between problematic smartphone use and depressive symptoms in Chinese adolescents
Source: Child Adolesc Psychiatry Ment Health. 2025 Jul 5;19:73. doi: 10.1186/s13034-025-00937-x (PMC12228315; doi:10.1186/s13034-025-00937-x)
Supplement: Supplementary file 1 — Additional file 1: Table S1. Adjacency matrix of the T1-T2 CLPN. Table S2. Adjacency matrix of the T2-T3 CLPN. Figure S1. Confidence intervals around edge weights for T1-T2 CLPN (left) and T2-T3 CLPN (right). Figure S2. Edge weight difference tests for T1-T2 CLPN and T2-T3 CLPN. Figure S3. Centrality difference tests for T1-T2 CLPN (a for Out Expected Influence, b for In Expected Influence) and T2-T3 CLPN (c for Out Expected Influence, d for In Expected Influence). Figure S4. The cross-lagged panel networks for T1-T2 (left) and T2-T3 (right) (including all autoregressive and cross-lagged edges). [file 13034_2025_937_MOESM1_ESM.docx]

**Table S1.** **Adjacency matrix of the T1-T2 CLPN. Independent variables (i.e., predictors) are in rows, and dependent variables are in columns.**

|  | P1 | P2 | P3 | P4 | P5 | P6 | P7 | P8 | P9 | M1 | M2 | M3 | M4 | M5 |
| --- | --- | --- | --- | --- | --- | --- | --- | --- | --- | --- | --- | --- | --- | --- |
| P1 | 1.00 | 1.00 | 0.99 | 1.00 | 1.00 | 1.00 | 1.00 | 1.00 | 1.00 | 0.96 | 1.00 | 1.00 | 1.00 | 1.00 |
| P2 | 1.01 | 1.08 | 1.00 | 1.00 | 1.00 | 1.00 | 1.00 | 1.00 | 1.08 | 1.11 | 1.05 | 1.00 | 1.00 | 1.00 |
| P3 | 1.00 | 1.00 | 1.14 | 1.03 | 1.00 | 1.00 | 1.00 | 1.00 | 1.00 | 0.93 | 1.00 | 1.00 | 1.00 | 1.00 |
| P4 | 1.05 | 1.00 | 1.02 | 1.04 | 1.00 | 1.07 | 1.02 | 1.00 | 1.01 | 1.11 | 1.05 | 1.06 | 1.06 | 1.20 |
| P5 | 1.04 | 1.01 | 1.19 | 1.15 | 1.24 | 1.03 | 1.00 | 1.03 | 1.02 | 0.92 | 1.00 | 1.00 | 1.00 | 0.96 |
| P6 | 1.06 | 1.00 | 1.00 | 1.00 | 1.00 | 1.10 | 1.04 | 1.00 | 1.02 | 1.00 | 1.00 | 1.00 | 1.00 | 1.00 |
| P7 | 1.03 | 1.06 | 1.03 | 1.12 | 1.04 | 1.01 | 1.19 | 1.05 | 1.00 | 1.01 | 1.00 | 1.00 | 1.00 | 0.97 |
| P8 | 1.07 | 1.09 | 0.94 | 1.00 | 1.00 | 1.00 | 1.00 | 1.04 | 1.04 | 1.00 | 1.00 | 1.00 | 1.00 | 1.00 |
| P9 | 1.00 | 1.11 | 1.00 | 1.00 | 1.00 | 1.04 | 1.00 | 1.03 | 1.12 | 1.00 | 1.00 | 1.00 | 1.00 | 1.00 |
| M1 | 1.00 | 1.00 | 1.00 | 1.05 | 1.00 | 1.00 | 1.00 | 1.00 | 1.00 | 1.36 | 1.01 | 1.02 | 1.09 | 1.07 |
| M2 | 1.05 | 1.11 | 1.09 | 1.04 | 1.04 | 1.10 | 1.05 | 1.05 | 1.00 | 1.00 | 1.51 | 1.09 | 1.00 | 1.15 |
| M3 | 1.02 | 1.00 | 1.14 | 1.00 | 1.01 | 1.01 | 1.03 | 1.01 | 1.00 | 1.12 | 1.11 | 1.48 | 1.09 | 1.00 |
| M4 | 1.04 | 1.00 | 1.06 | 1.07 | 1.02 | 1.02 | 1.06 | 1.01 | 1.03 | 1.11 | 1.04 | 1.05 | 1.62 | 1.10 |
| M5 | 1.00 | 1.00 | 1.00 | 1.00 | 1.00 | 1.00 | 1.00 | 1.00 | 1.00 | 1.05 | 1.00 | 1.00 | 1.00 | 1.30 |

**Table S2.** **Adjacency matrix of the T2-T3 CLPN. Independent variables (i.e., predictors) are in rows, and dependent variables are in columns.**

|  | P1 | P2 | P3 | P4 | P5 | P6 | P7 | P8 | P9 | M1 | M2 | M3 | M4 | M5 |
| --- | --- | --- | --- | --- | --- | --- | --- | --- | --- | --- | --- | --- | --- | --- |
| P1 | 1.08 | 1.12 | 1.03 | 1.08 | 1.00 | 1.07 | 1.05 | 1.02 | 1.05 | 1.00 | 1.00 | 1.00 | 1.00 | 1.00 |
| P2 | 1.23 | 1.38 | 1.11 | 1.15 | 1.07 | 1.18 | 1.12 | 1.10 | 1.00 | 1.00 | 1.00 | 1.00 | 1.00 | 1.00 |
| P3 | 1.19 | 1.21 | 1.35 | 1.27 | 1.33 | 1.08 | 1.19 | 1.13 | 1.06 | 1.00 | 1.00 | 1.02 | 1.00 | 1.00 |
| P4 | 1.00 | 0.97 | 1.00 | 1.00 | 1.00 | 1.00 | 1.06 | 1.00 | 1.00 | 1.07 | 1.09 | 1.04 | 1.08 | 1.22 |
| P5 | 1.06 | 0.95 | 1.00 | 1.00 | 1.00 | 1.00 | 1.00 | 1.00 | 1.00 | 1.00 | 0.94 | 1.00 | 1.00 | 1.00 |
| P6 | 1.14 | 1.13 | 1.10 | 1.15 | 1.08 | 1.23 | 1.11 | 1.11 | 1.25 | 1.00 | 1.14 | 1.06 | 1.00 | 1.00 |
| P7 | 1.04 | 0.93 | 1.02 | 1.04 | 1.08 | 1.03 | 1.04 | 1.00 | 1.00 | 1.04 | 1.05 | 1.04 | 1.03 | 1.00 |
| P8 | 0.85 | 1.01 | 1.00 | 1.00 | 1.00 | 1.00 | 1.00 | 1.06 | 1.00 | 1.00 | 1.01 | 1.00 | 1.00 | 1.00 |
| P9 | 1.09 | 1.14 | 1.08 | 1.04 | 1.04 | 1.00 | 1.06 | 1.01 | 1.19 | 1.00 | 1.00 | 1.00 | 1.00 | 1.00 |
| M1 | 1.05 | 1.03 | 1.04 | 1.02 | 1.03 | 1.03 | 1.04 | 1.01 | 1.02 | 1.41 | 1.08 | 1.09 | 1.09 | 1.08 |
| M2 | 1.00 | 1.00 | 1.02 | 1.05 | 1.09 | 1.00 | 1.02 | 1.03 | 1.00 | 1.02 | 1.46 | 1.00 | 1.01 | 1.19 |
| M3 | 1.09 | 1.13 | 1.04 | 1.04 | 1.00 | 1.07 | 1.09 | 1.08 | 1.07 | 1.00 | 1.09 | 1.45 | 1.08 | 1.00 |
| M4 | 1.00 | 1.03 | 1.01 | 1.00 | 1.00 | 1.04 | 1.01 | 1.00 | 1.00 | 1.19 | 1.08 | 1.07 | 1.72 | 1.10 |
| M5 | 1.00 | 1.01 | 1.01 | 1.03 | 1.04 | 1.00 | 1.01 | 1.00 | 1.00 | 1.01 | 1.06 | 1.00 | 1.00 | 1.35 |


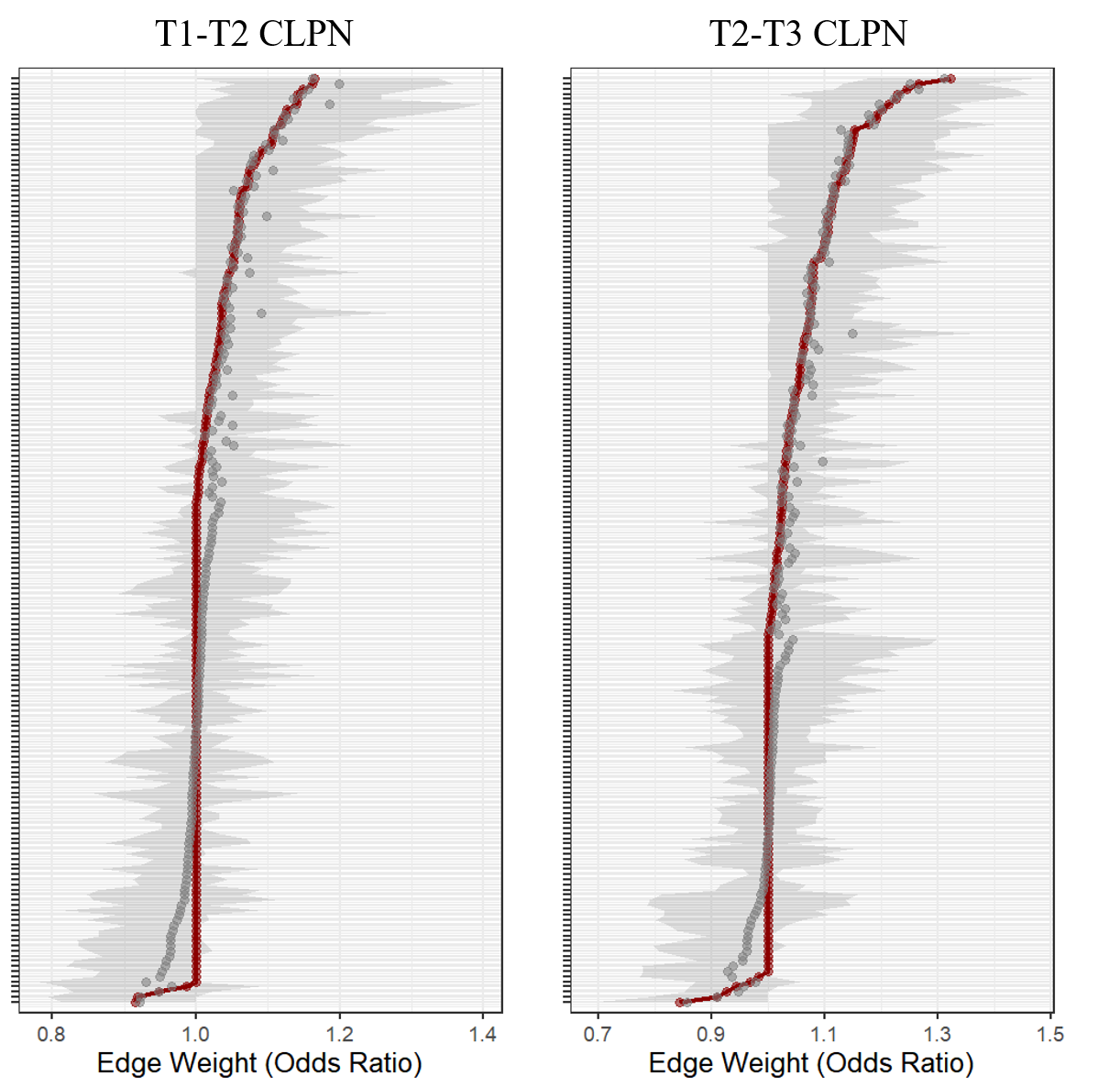


**Figure** **S1. Confidence intervals around edge weights for T1-T2** **CLPN and T2-T3 CLPN.**


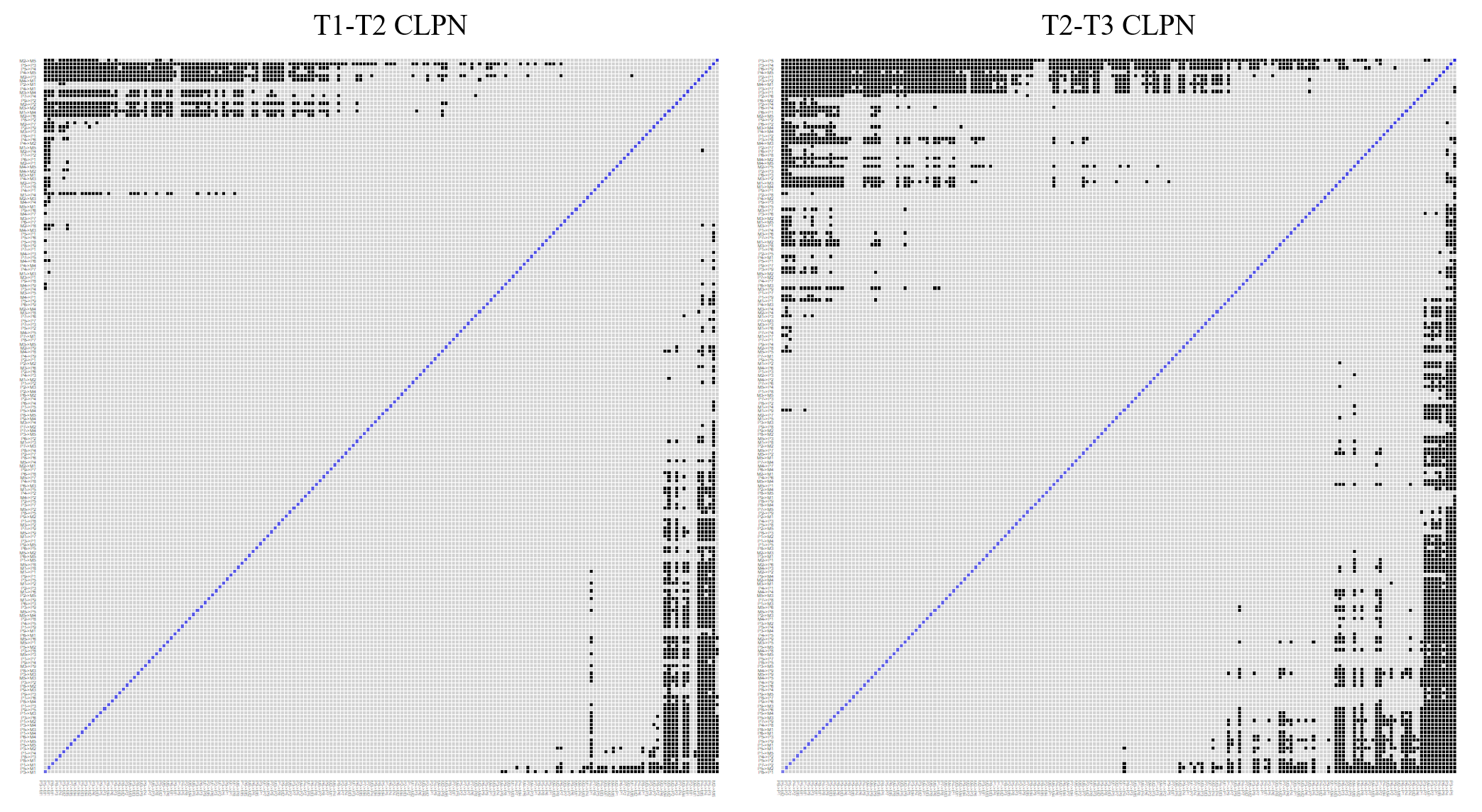


**Figure S2. Edge weight difference tests for T1-T2 CLPN and T2-T3 CLPN.**


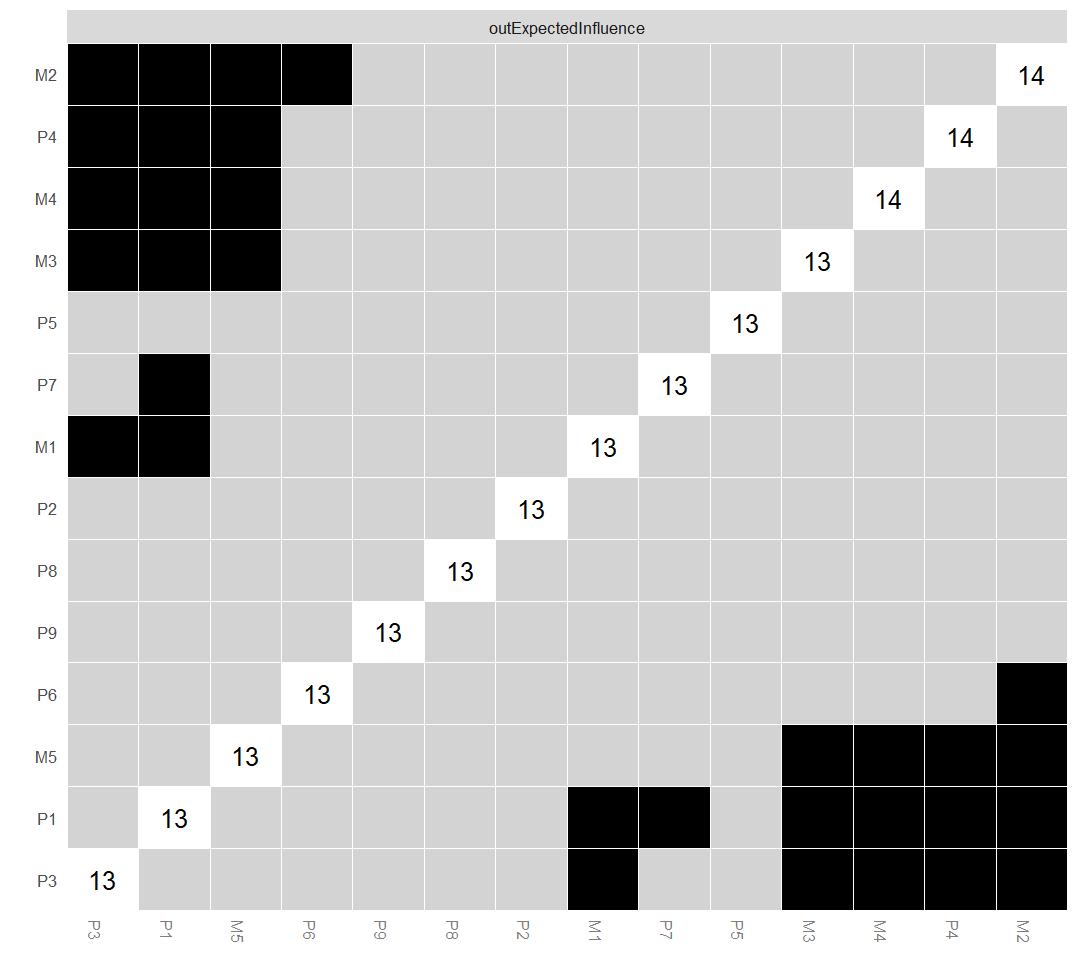


a


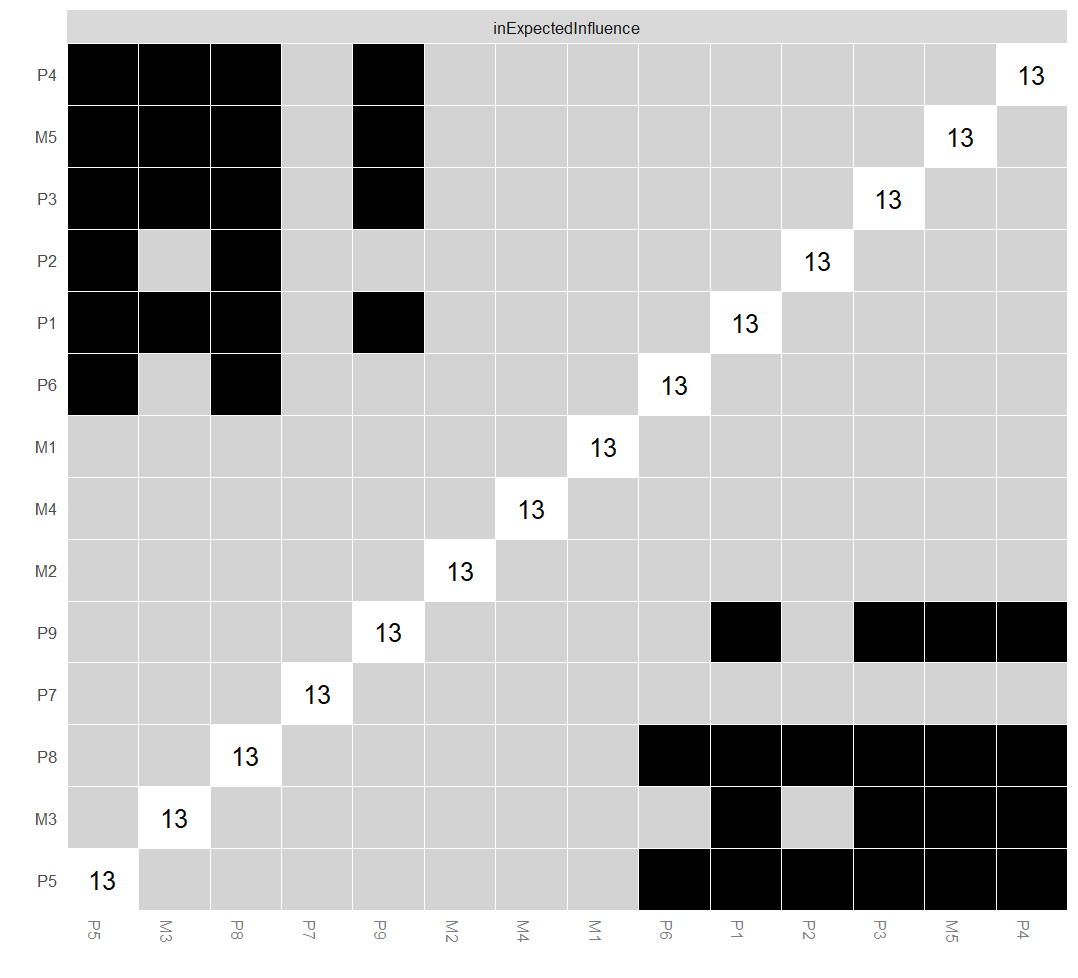


b


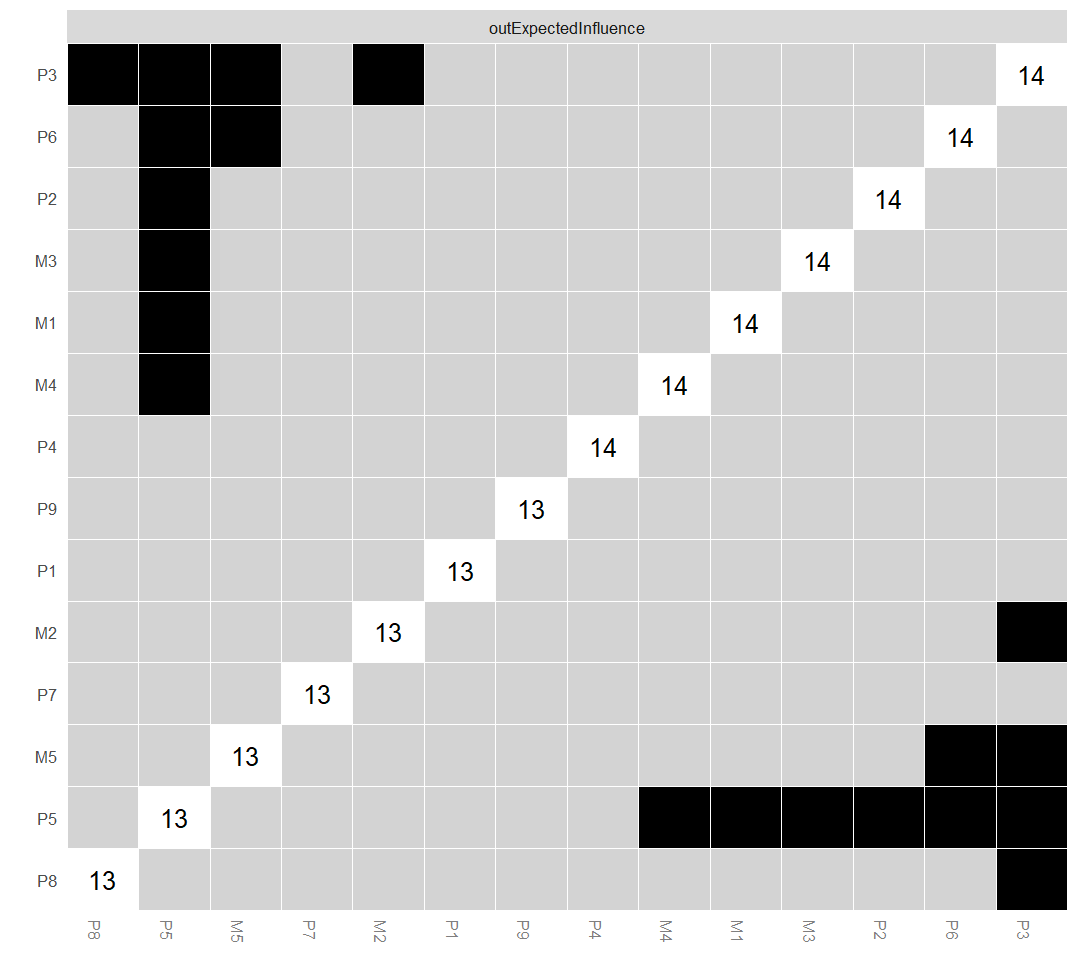


c


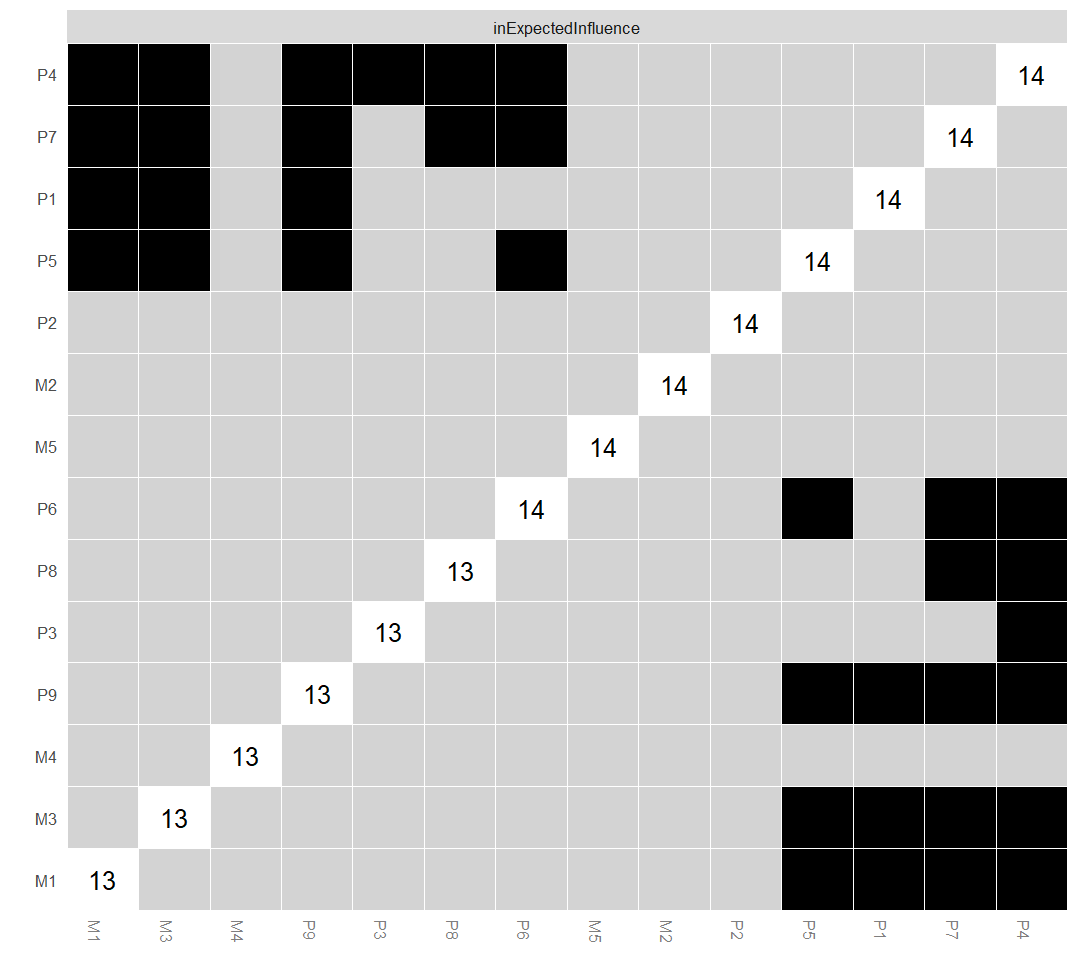


d

**Figure S3**. **Centrality difference tests for T1-T2 CLPN (a for Out Expected Influence, b for In Expected Influence) and T2-T3 CLPN (c for Out Expected Influence, d for In Expected Influence).**


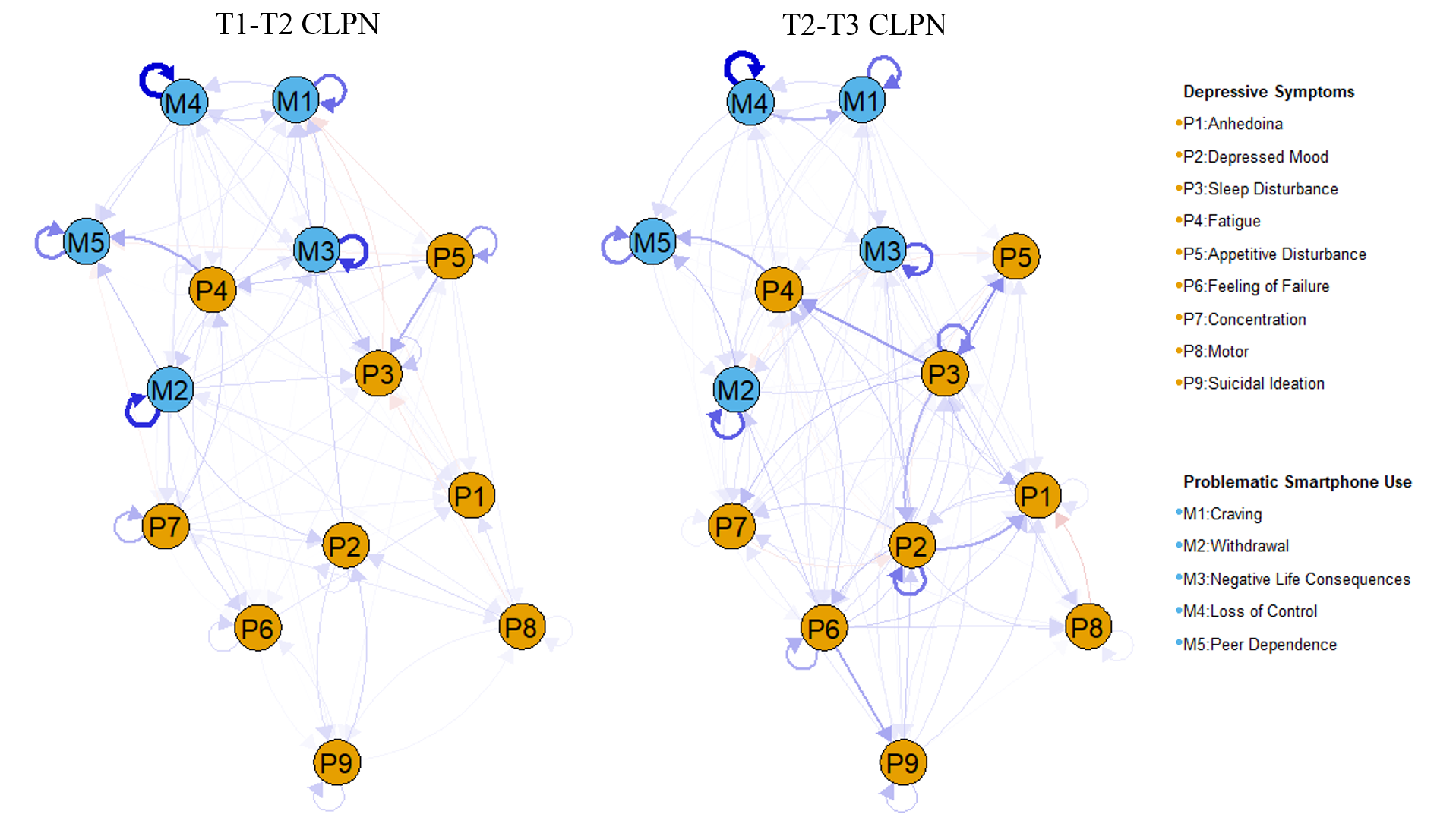


**Figure S4.** **The cross-lagged panel networks for T1-T2 and T2-T3 (including all autoregressive and cross-lagged edges)**
